# Phase II study of high-dose medroxyprogesterone acetate in advanced malignant melanoma.

**DOI:** 10.1038/bjc.1989.201

**Published:** 1989-06

**Authors:** R. Becher, O. Kloke, K. HÃ¶ffken, M. E. Scheulen, U. B. Wandl, H. Bojar, C. G. Schmidt

**Affiliations:** Innere Klinik und Poliklinik (Tumorforschung), Westdeutsches Tumorzentrum, Essen, Federal Republic of Germany.


 © The Macmillan Press Ltd., 1989

SHORT COMMUNICATION

### Phase II study of high-dose medroxyprogesterone acetate in advanced malignant melanoma

R. Becher, O. Kloke, K. Höffken, M.E. Scheulen, U.B. Wandl, H. Bojar & C.G. Schmidt

*Innere Klinik und Poliklinik (Tumorforschung), Westdeutsches Tumorzentrum, Hufelandstrasse 55, 4300 Essen 1, Federal Republic of Germany.*

It has been shown that a small number of patients with advanced malignant melanoma (MM) respond to additive hormonal treatments such as the anti-oestrogen tamoxifen (Karakousis *et al.*, 1980). Receptor studies have shown that the expression of significant amounts of oestrogen receptors is rare in MM. However, glucocorticoid receptors are a common finding (Hawkins *et al.*, 1980; Bhakoo *et al.*, 1981; Bojar *et al.*, 1982). The observation that medroxyprogesterone acetate (MPA) binds to the glucocorticoid receptor in the same way as dexamethasone, led to a phase II study to assess the therapeutic relevance of the high glucocorticoid receptor levels in MM.

Eighteen outpatients with histologically confirmed MM and measurable and progressive lesions, who were not amenable to curative surgery were treated with high-dose MPA. The patients had a performance status according to the Karnofsky index above or at 70%. The characteristics of patients are shown in Table I.

All patients received 1 g MPA per day as intramuscular injection on days 1–10 and 200 mg MPA t.i.d. orally thereafter. This treatment schedule was adapted from a treatment schedule for advanced breast cancer and yields MPA serum levels well above 100 ng ml$^{-1}$ (Miller *et al.*, 1985). Treatment was continued as long as disease stabilisation or remission was achieved. The criteria for response were as follows. Tumour progression was defined as an increase of tumour size of more than 25% of any measurable lesion or the appearance of new lesions. Partial response was defined as a reduction of tumour size of more than 50% of every measurable lesion without appearance of new lesions. Complete response was defined as disappearance of all measurable lesions.

All patients were evaluable for response. Duration of treatment ranged from 13 days to 10 months (median 50 days). A stabilisation of disease was observed in two patients for 3 months. There were no objective responses. Toxicity was moderate and similar to that reported for high-dose MPA treatment in breast cancer. Weight gain of more than 3 kg was observed in three patients.

**Table I** Pretreatment characteristics of patients

| *Characteristic* | *No. of patients* |
|---|---|
| Entered / evaluated | 18 / 18 |
| Pretreatment | |
| None | 9 |
| Tamoxifen | 7 |
| Chemotherapy | 2 |
| Metastatic sites | |
| Lung | 7 |
| Liver | 6 |
| Lymph nodes | 12 |
| Only one site | 7 |
| More than one site | 10 |

Median age in years (range): 45 (12–72).

Our experience with high-dose medroxyprogesterone acetate was negative and similar to that reported by Creagan *et al.* (1982), who treated 20 patients with progesterone megestrol acetate.

We conclude that HD-MPA has only little or no tumour-reductive effect in advanced MM (response less than 20% with 95% confidence). Thus, the high levels of glucocorticoid receptors found in the majority of melanoma specimens and their binding affinity to MPA seem not to be of therapeutic relevance. In addition, high-dose MPA treatment is associated with a higher level of toxicity, as compared to tamoxifen, which is able to induce incidental tumour reductions (Karakousis *et al.*, 1980).

### References

BHAKOO, H.S., MILHOLLAND, R.J., LOPEZ, R., KARAKOUSIS, C. & ROSEN, F. (1981). High incidence and characterization of glucocorticoid receptors in human malignant melanoma. *JNCI*, **66**, 21.

BOJAR, H., STUHLDREIER, B., BECHER, R., KOLDOVSKY, U., GOERZ, G., MERK, H. & STAIB, W. (1982). Gradient centrifugation analysis of steroid hormone binding in human malignant melanoma. *Anticancer Res.*, **2**, 245.

CREAGAN, E.T., SCHUTT, A.J., AHMANN, D.L. & GREEN, S.J. (1982). Phase II study of high-dose megestrol acetate in patients with advanced malignant melanoma. *Cancer Treat. Rep.*, **66**, 1239.

HAWKINS, E.F., HORN, D. & MARKLAND, F.S. (1980). Receptor for glucocorticoids in RPMI 3460 melanoma cells. *Cancer Res.*, **40**, 2174.

KARAKOUSIS, C.P., LOPEZ, R.E., BHAKOO, H.S., ROSEN, F., MOORE, R. & CARLSON, M. (1980). Estrogen and progesterone receptors and tamoxifen in malignant melanoma. *Cancer Treat. Rep.*, **64**, 819.

MILLER, A.A., BECHER, R. & SCHMIDT, C.G. (1985). Langzeitverlauf der Pharmakonzentration während der Therapie mit Medroxyprogesteronacetat. In *Medroxyprogesteronacetat (MPA) in der Onkologie*, C.G. Schmidt *et al.* (eds) p. 53. Schattauer: Stuttgart and New York.

Correspondence: R. Becher.
Received 9 January 1989, and in revised form, 25 January 1989.


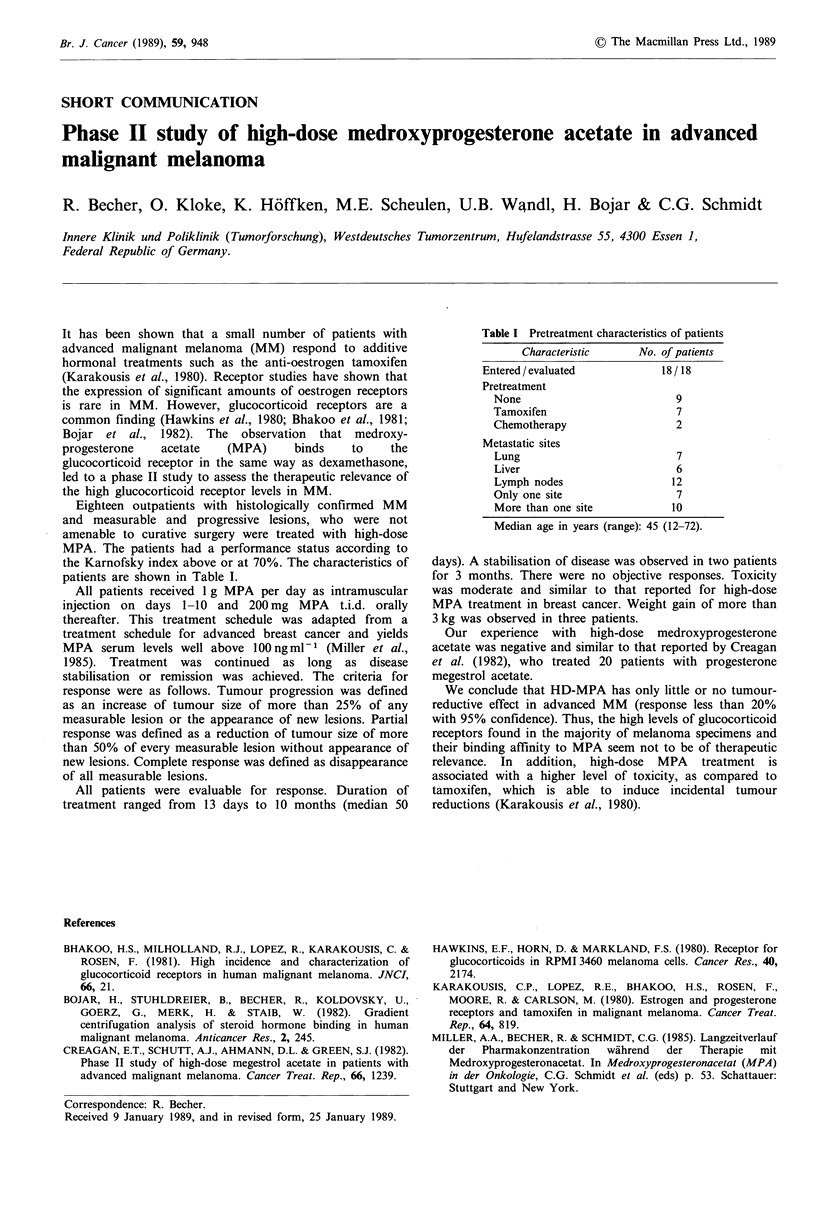


## References

[OCR_00096] Bhakoo H. S., Milholland R. J., Lopez R., Karakousis C., Rosen F. (1981). High incidence and characterization of glucocorticoid receptors in human malignant melanoma.. J Natl Cancer Inst.

[OCR_00102] Bojar H., Stuhldreier B., Becher R., Koldovsky U., Goerz G., Merk H., Staib W. (1982). Gradient centrifugation analysis of steroid-hormone binding in human malignant melanoma.. Anticancer Res.

[OCR_00108] Creagan E. T., Schutt A. J., Ahmann D. L., Green S. J. (1982). Phase II study of high-dose megestrol acetate in patients with advanced malignant melanoma.. Cancer Treat Rep.

[OCR_00117] Hawkins E. F., Horn D., Markland F. S. (1980). Receptor for glucocorticoids in RPMI 3460 melanoma cells.. Cancer Res.

[OCR_00122] Karakousis C. P., Lopez R. E., Bhakoo H. S., Rosen F., Moore R., Carlson M. (1980). Estrogen and progesterone receptors and tamoxifen in malignant melanoma.. Cancer Treat Rep.

